# Supplementary material for: P-TRAP: a Panicle Trait Phenotyping tool
Source: BMC Plant Biol. 2013 Aug 29;13:122. doi: 10.1186/1471-2229-13-122 (PMC3848748; doi:10.1186/1471-2229-13-122)
Supplement: Additional file 7 — P-TRAP source code. The Netbeans project that contains the source code of the application. [file 1471-2229-13-122-S7.zip › javadoc.zip!/javadoc/org-bioalg-riceprojecttype/org/bioalg/riceprojecttype/package-tree.html]

org.bioalg.riceprojecttype Class Hierarchy (RiceProjectType)


JavaScript is disabled on your browser.


- Package
- Class
- Tree
- Deprecated
- Index
- Help

*org.bioalg.riceprojecttype 1.0*

- Prev
- Next

- Frames
- No Frames

- All Classes

# Hierarchy For Package org.bioalg.riceprojecttype

## Class Hierarchy

- java.lang.Object
  - org.bioalg.riceprojecttype.RiceProject (implements org.netbeans.api.project.Project)
  - org.bioalg.riceprojecttype.RiceProjectFactory (implements org.netbeans.spi.project.ProjectFactory)

- Package
- Class
- Tree
- Deprecated
- Index
- Help

*org.bioalg.riceprojecttype 1.0*

- Prev
- Next

- Frames
- No Frames

- All Classes
